# Is There a Role for an Oral Physician in Diagnosing Undiagnosed Depression?

**DOI:** 10.21315/mjms2019.26.1.15

**Published:** 2019-02-28

**Authors:** Ramasamy Chidambaram

**Affiliations:** Department of Prosthodontics, Faculty of Dentistry, AIMST University, Jalan Bedong-Semeling, 08100 Bedong, Kedah, Malaysia

Dear Editor,

The topic receiving a great attention nowadays is stress. Chronic stress leads to depression. It is a mental illness, which most of us dismiss as a matter of concern. It has been estimated that by 2030, the most disabling disease would be depression, scoring the highest loss of working hours. In Malaysia, it is sad to know that at least 40% population suffer from mental health issues (1). On a positive side, Malaysia marked the World Health Day 2017 with a focus on depression. Conjointly, the Malaysian Mental Health Association—a forefront organisation in Malaysia—has been serving since 1967 in organising intensive courses and public talks to cope the mental illness (2). If there has been a great progress in the anti-depression programme in Malaysia then why there is a gradual rise in the new admissions. Are we missing something here? My hypothesis is that the undiagnosed cases of depression are probably the hidden reasons for this increase.

It is weird to expect a dental patient to share his/her experience of depression with an oral physician (OP). In most cases, physical problems, such as head-ache and constant fatigue, are reported to a family physician (FP). Thus, is it possible for an OP to diagnose undiagnosed depression?

A bi-directional association between oral and mental health has been reported recently (3). In one way, oral health might be a risk factor for depression, while many psychiatric conditions, such as severe mental illness, affective disorders, and eating disorders, could lead to dental disease. However, the new investigations suggest a potential strategy for the OPs to re-emphasise the importance of an oral-systemic link. There is no depression test. It is based on a careful observation and thorough medical history that includes questions about psychiatric or mental health-care, substance use, current medication, dental fear, and depression or anxiety. This may reveal underlying symptoms of depression like loss of appetite/weight, lack of interest, insomnia, slowed speech and suicidal thoughts (4). It has been suggested that when patients with such complaints are being treated, the possibility of depression should be considered. Furthermore, depressed patients are more prone to dry mouth, dental caries, and periodontal disease due to neglecting oral-care (3). If left untreated, dental diseases can lead to teeth loss, as people with severe mental illness have 2.7 times higher possibility of losing all teeth than the healthy population (3). If an OP observes that a patient is suffering from depression, his good rapport will help him to make the patient understand the necessity of a psychological check-up and eventually can be referred to a psychiatrist. In most cases, referral to the FP may be better accepted by the patient; however, the availability of both options provides more flexibility in dealing with individual situations. Even OPs can counsel a patient because they may be the first to identify undiagnosed depression on the basis of oral signs and depressive symptoms. In conclusion, everyday health problems are receiving threats from new links (5, 6); therefore an increased focus on the oral-health of people with a psychiatric illness should be given.
